# Tephritid Fly Abundance and Inflorescence Herbivory on a High Alpine Thistle Vary with Environment

**DOI:** 10.3390/insects17101015

**Published:** 2026-10-01

**Authors:** Raven A. Reville, Emily L. Schultz, Svata M. Louda

**Affiliations:** 1 Department of Biological Sciences, Ecosystem Science and Stewardship Program, Colorado Mountain College, Breckenridge, CO 80424, USA; 2 School of Biological Sciences, University of Nebraska, Lincoln, NE 68588, USA; svata.louda@unl.edu

**Keywords:** tephritid flies, *Cirsium*, pre-dispersal seed predation, alpine herbivory, habitat partitioning, guild structure, floral herbivory, seed reproductive success

## Abstract

Little is known about insect–plant interactions in high alpine environments. We studied the insect herbivore feeding guild in inflorescences of *Cirsium funkiae* (Asteraceae), an alpine thistle endemic to the southern Rocky Mountains, USA. We asked whether site (open talus above treeline, mid-elevation wet meadow, or lower-elevation forest edge just below treeline) and plant characteristics affect insect abundance, guild composition, and intensity of herbivory. We dissected flower heads collected at season’s end to quantify fly occurrence and measure herbivory damage. Three tephritid flies—*Chaetostomella undosa*, *Paracantha gentilis*, and *Terellia occidentalis*—strongly dominated the herbivore guild. All three species differed in abundance and composition across habitat sites. *Chaetostomella* dominated in the high, open talus habitat; *Terellia* peaked in the mid-elevation meadow site; and *Paracantha* dominated in the lower forest edge site. While *Terellia* abundance was strongly associated with flower head diameter, abundances of *Paracantha* and *Chaetostomella* were only weakly associated with flower head size, suggesting species-specific flower-head selection. *Paracantha* and *Terellia* showed elevated co-occurrence at the mid-elevation alpine meadow site, indicating shared distributional preferences likely related to their occurrence on thistles at lower elevations in this region. Unexpectedly, seed damage was extremely high (91.5–98.5%) across all three sites and highest in the characteristic habitat, open high talus slopes. Despite uniformly high seed damage, the low Forest Edge plants had significantly more viable seeds than did the High site plants (8.2% vs. 1.6%), and the difference was associated with differences in guild composition. These findings demonstrate that tephritid flies dominate floral herbivory on the high alpine *Cirsium funkiae*, that site differences can shape fly feeding guild structure, and that they have a measurable negative impact on plant seed reproductive success in high alpine environments.

## Simple Summary

Insect herbivores can influence plant survival and reproduction, yet we know little about these interactions in alpine environments. This study examined insects associated with *Cirsium funkiae*—a rare thistle found only in the southern Rocky Mountains—in southern Colorado, USA, growing across three different habitat types: an open rocky slope above treeline, a wet meadow, and a sheltered forest edge; the habitats also differed in elevation. When we dissected flower heads, we found that rather than a diverse feeding guild, the flower heads were occupied almost exclusively by fruit flies belonging to the tephritid group. Three species were identified, each one showing preference for a particular site, suggesting they have developed distinct ecological patterns of usage despite sharing the same host plant. The number of flies on individual flower heads was also influenced by the size of the flower head, with larger heads harboring more flies of certain species. Despite high levels of seed destruction at all sites, plants at the low, forest edge site produced more viable seeds than did plants on the high, open rocky slope, a difference linked to which fly species were predominant. These findings suggest that in alpine environments, feeding guilds can be well organized, and the insects present in a site can have meaningful negative consequences for plant reproduction.

## 1. Introduction

Insect herbivores are often important drivers of plant performance and population dynamics across a wide range of ecosystems, influencing plant reproductive success and demography, as well as plant density and distribution [1,2,3,4,5,6,7]. In alpine and subalpine systems, where short growing seasons and harsh environmental conditions already limit plant performance [8], the additional pressure of insect herbivory could be significant. Despite this, the occurrence, dynamics and outcomes of insect herbivory on plants in alpine habitats remain understudied [9,10], especially herbivory by internal-feeding floral and seed herbivores.

The prevailing expectation has been that insect herbivory should decrease with increasing elevation, given the increase in harshness of growing conditions. In fact, a general decrease in insect diversity and abundance at higher altitudes is often reported [11,12]. However, recent evidence from mountain systems around the world suggests a more complicated story. In some systems and under some conditions, insect herbivory has been shown to increase rather than decrease with elevation. For example, this was observed in open alpine habitats, where surface temperatures can exceed air temperatures, creating warm microenvironments that support active insect herbivore communities [12,13]. These findings suggest that elevation per se may not be as strong or consistent a predictor of herbivore feeding intensity as expected. Other, as yet underexplored factors may provide alternate explanations for the composition, abundances, and behavior in some insect assemblages on plants in the high alpine.

Microhabitat conditions, localized environmental characteristics experienced by individual plants, including soil substrate type, soil moisture availability, solar exposure, and surrounding vegetation, have emerged as important in both plant performance and herbivore community structure [14]. Plants growing at the same elevation but in different microhabitats can experience dramatically different sets of herbivores and consequent levels of damage [11]; in that case, high-elevation plant populations in the open were more heavily impacted by insect herbivores than were those in the forest at the same elevation. Substrate type also has been shown to influence plant dynamics independent of elevation, including in our study region in the Sangre de Cristo Range, Colorado, USA [15].

**Figure 1:**
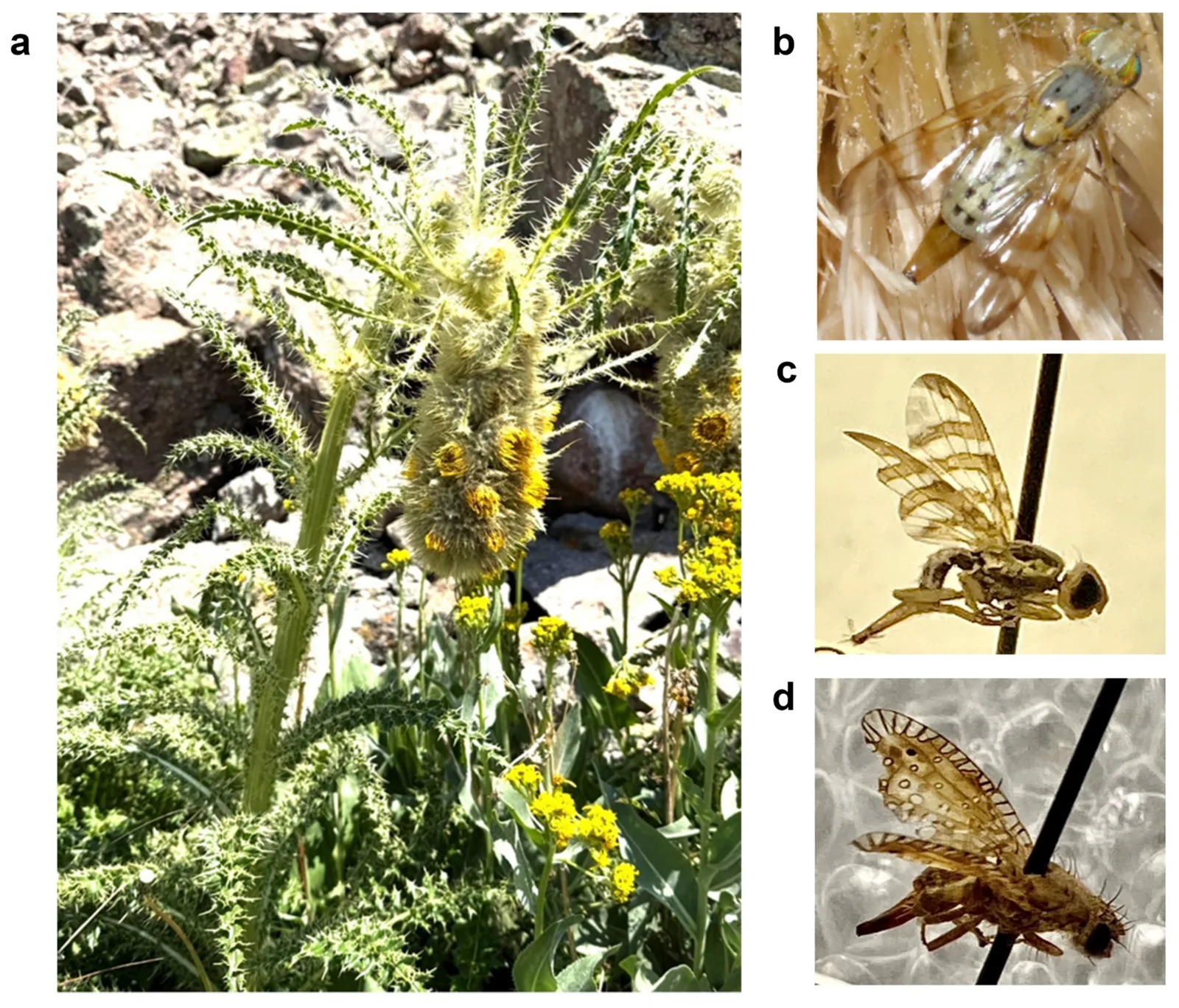
(**a**) *Cirsium funkiae* in its typical high-elevation rocky talus habitat, showing flowering stem size, aggregation of flower heads, composed of discoid, yellow florets, in a compacted, nodding terminal cluster; (**b**) *Terellia occidentalis* female; (**c**) *Chaetostomella undosa* female; and (**d**) *Paracantha gentilis* female. *Paracantha gentilis* and *Terellia occidentalis* are oligophagous on multiple *Cirsium* species, and they have broad geographic and elevational distributions across the western USA [16,17]. Plant photo by Kevin Sloan; insect photos by S. M. Louda.

We studied the prominent insect floral and seed herbivores, which were tephritid flies, and their effects on *Cirsium funkiae*, quantifying the internal feeding on reproductive tissues, ovules, and developing seed among three alpine sites (Figure 1). This native thistle, a relatively uncommon, high-alpine species endemic to the southcentral Rocky Mountains, was only recently identified as genetically distinct, after more than 150 years of inclusion in *C. scopulorum* [18]. Its typical habitat is dry, open, high alpine talus slopes above treeline.

The Tephritidae are characteristic pre-dispersal flower- and seed-feeders on *Cirsium* spp. thistles throughout North America; they oviposit directly into developing tissues, usually immature flower heads, and consume developing ovules and seeds of the heads as larvae [19]. Their impact on host plant reproduction and dynamics can be severe. For example, in prairie grasslands, pre-dispersal seed loss imposed on native *C. canescens* populations, especially by two species of native tephritid flies, decreased seed reproductive success, limited plant recruitment, and significantly reduced the population growth rate, lambda [4,20]. The tephritids significantly limit seed reproductive success of *C. canescens* even in montane grassland populations at the western edge of the species distribution [21]. However, despite their known ecological importance, the occurrence and feeding damage of tephritids on high alpine *Cirsium* spp. in North America has not been investigated. Notably, two of the three tephritid species found in this study are oligophagous across multiple *Cirsium* species and have broad geographic and elevational distributions across the western United States [16,17]. The host range for the third, *Chaetostomella undosa*, is less well documented, though it has been recorded on multiple *Cirsium* species in the western United States [16,22]. This prevalence makes this gap in knowledge particularly surprising.

Although the typical habitat for *C. funkiae* is dry, exposed, talus rock slopes above treeline, occasionally plants are observed in wet, snowmelt drainage meadows and along forest edge habitats at or just below treeline. Environmental conditions associated with these wetter, less exposed habitats differ significantly from those in the usual dry, open rock habitat. This variation in microhabitat occupancy by *C. funkiae* provided an opportunity to ask whether, and if so, how habitat variation influenced both tephritid guild structure and their herbivory on a native high alpine host plant.

In this study, we asked: (1) do abundance and tephritid guild composition within flower heads vary between habitats?; (2) does tephritid species co-occurrence vary between habitats?; (3) does habitat-related variation in plant traits influence tephritid species abundances?; and (4) does variation in herbivore numbers or feeding guild composition predict differences in the intensity of herbivory and subsequent plant seed reproductive success between habitats? Understanding how tephritid guild composition and herbivory intensity vary with exposure and elevation differences in this system can provide predictions for understanding how insect herbivore—plant interactions are structured in other understudied alpine systems.

## 2. Materials and Methods

### 2.1. Study Sites

This study was conducted along the South Crestone Lake Trail in the Sangre de Cristo Range, southcentral Rocky Mountains, Colorado, USA. The Sangre de Cristo Range is characterized by steep grades, with highly variable substrate conditions including granitic bedrock and talus [23,24]. The climate is typical of southern Rocky Mountain alpine systems with a short growing season (late May–mid-September); it is characterized by: late-season residual snowpack in spring at high elevations; high solar radiation; large daily temperature fluctuations, including in the growing season; and high spatial variation in moisture, related to wind, exposure, and orographic effects [25].

The study sites represented the three main habitats in which *Cirsium funkiae* occurred in this area. The High site (38.014° N, 105.627° W; 3597 m) was a dry, rocky, open alpine area that was representative of the reported predominant *C. funkiae* habitat. The High site was characterized by rocky talus substrate with large boulders, extremely high solar exposure, and very sparse vegetation. The Meadow site (38.012° N, 105.633° W; 3414 m) was a wet drainage meadow adjacent to South Crestone Creek, with high soil moisture, strong midday sun exposure, and mixed grassland-shrub vegetation. The Forest Edge site (38.008° N, 105.644° W; 3231 m) also had relatively moist soil, but only moderate, midday direct insolation, typical of the grassland-shrub to mixed evergreen forest ecotone; it had intermediate soil moisture and the least solar exposure of the three sites. Thus, the sites differed in solar radiation, topographically driven soil moisture, vegetation, and elevation.

### 2.2. Field Data, Flower Head Collection, and Dissection

*Cirsium funkiae* plants, brought to our attention by Robert Dorenfeld, were measured in situ (rosette diameter, number of flowering stems, and flowering stem heights). After photographs of the site and plant were taken, the main flowering stem per plant was collected for flower head dissection (*n* = 9 plants with 317 flower heads: High site, 141 heads; Meadow site, 84 heads; and Forest Edge site, 92 heads). All samples were collected 14 August 2025 near the end of the growing season, by Kevin Sloan as part of a Citizen Science Project on Alpine Thistle—Insect Interactions. Since collection was limited to a single date and 9 plants, there are temporal and spatial constraints that should be considered when interpreting the results. The main portion of the pendulous flowering stem, a cluster with the majority of heads initiated, was removed and placed into a paper bag, which was sealed; samples were transported to the lab and stored at room temperature until examined. There are no voucher specimens of the plants deposited in any collections, but voucher specimens of the insects have been deposited at the University of Colorado Museum of Natural History, Entomology Division, Boulder, CO, USA, as the Louda collection.

Each flower head was bisected, photographed, and dissected to quantify insect occurrence, insect feeding damage, and seed reproductive success. All insects found were recorded, including number and description of all stages of the tephritid flies present, and the number and description of other insects present. All evidence of insect occurrence was recorded. This included dead or desiccated specimens of varying life stages, all of which were counted for the analyses. The Tephritidae were identified by Dr. David H. Headrick of California Polytechnic State University, using pinned specimens on hand. The variables recorded for each flower head were: phenological number, developmental stage reached, and mean diameter (mm) attained; presence of stem damage, tunneling or mealy texture; and receptacle condition, including shape in profile, and proportions of stem, stem-receptacle area, and receptacle surface damaged by insect feeding. Identifiable seeds or ovules remaining in each head were counted and sorted by condition: undamaged filled (“good”) seeds, possibly undamaged filled seeds, insect-damaged filled seeds, and aborted ovules; aborted ovules were shriveled, darkened, and many had evidence of insect feeding. We then calculated the proportion of filled, potentially viable seeds as the ratio of good (filled, undamaged) seeds to total seeds remaining (conservative estimate), as well as the proportion of seeds and ovules damaged by the insect feeding.

### 2.3. Environmental Variables and Characteristics

Environmental variables were extracted for each site using ArcGIS Pro version 3.7 [26]. Topographic Wetness Index (TWI) and solar radiation were calculated from a 10 m resolution digital elevation model (DEM). TWI is a measure of topographic moisture potential, with higher values indicating wetter, moisture-retaining terrain. Solar radiation values modeled annual radiation in watt-hours per square meter (Wh/m^2^). Both variables were extracted using three spatial buffer sizes: 15 m, 25 m, and 50 m. Vegetation cover data were derived from the 2024 National Land Cover Database (NLCD) [27] at 30 m resolution using a 50 m buffer, and land cover classes were summarized as proportional cover of each NLCD category within each site buffer.

**Figure 2:**
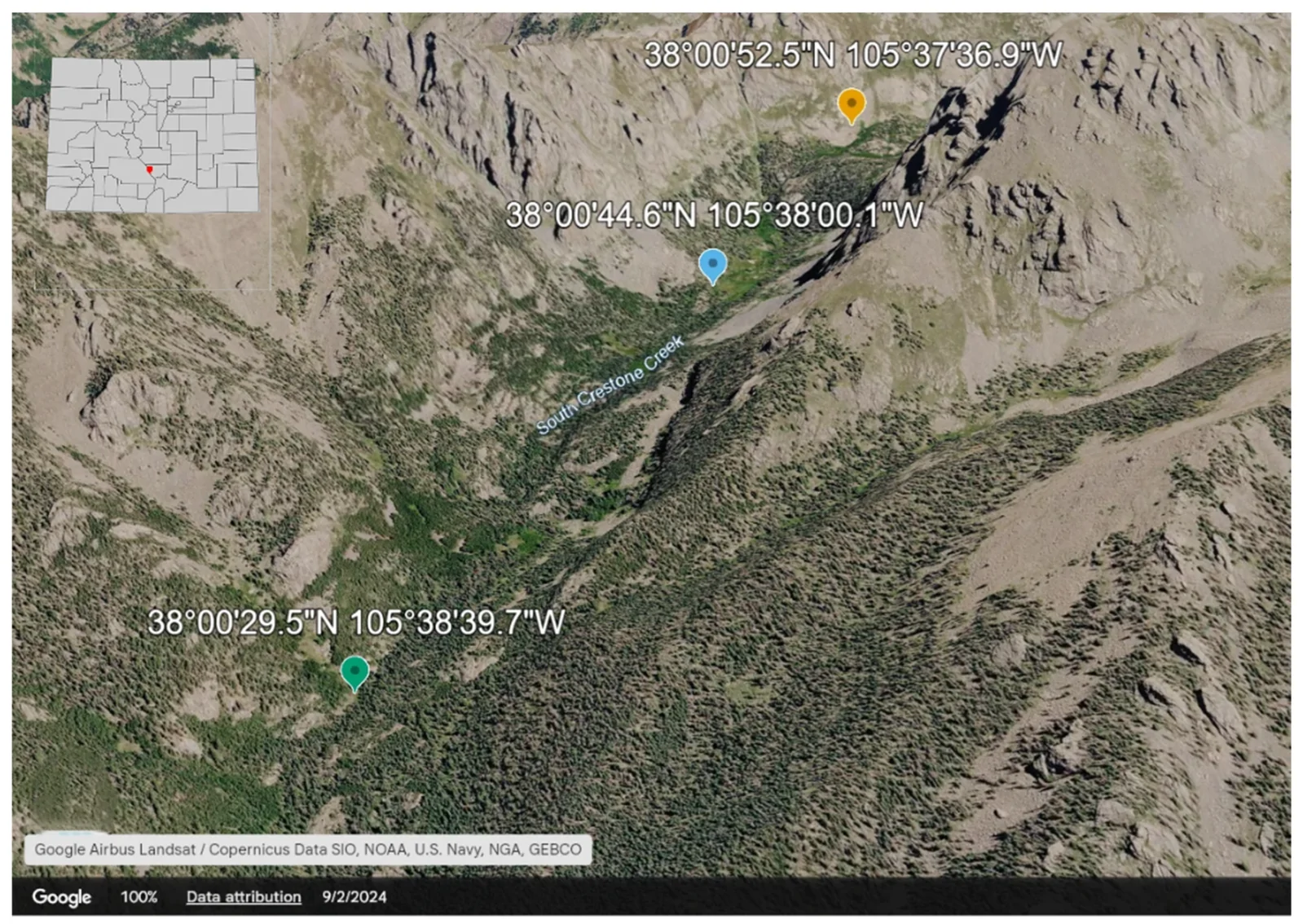
The locations of the sites representing the three habitats along the South Crestone Lake Trail in the Sangre de Cristo Range, southcentral Rocky Mountains, Colorado, USA (centered at 38°00′41.0″ N, 105°38′08.3″ W): Yellow = High site (3597 m), typical habitat; blue = Meadow site (3414 m) along Crestone Creek; and green = Forest Edge site (3231 m) at the conifer—meadow ecotone. The red point on the map of Colorado represents the site’s location within the state. Overlay vector mapping out state boundaries generated from open-source shapefiles provided by the Colorado Department of Public Health and Environment (CDPHE) [28] and stylized using Esri ArcGIS Pro software version 3.7. Data layers and thematic mapping were generated using Esri ArcGIS Pro software. Image generated from a camera altitude of approximately 1607 m using the Google Earth web application [29]. Satellite imagery incorporates data captured between 1 January 2021 and 22 August 2025. Data and imagery providers: Google, Airbus, Landsat/Copernicus, and Data SIO, NOAA, U.S. Navy, NGA, GEBCO. This figure is excluded from the CC BY 4.0 license of this article and is reproduced under Google’s Geo Guidelines.

Sites varied greatly in environmental characteristics (Figure 2). High site was characterized by rock talus slopes surrounded by open low shrub and grassland vegetation (61.5% shrub/scrub and 38.5% grassland within a 50 m buffer). The High site also had moderate mean solar radiation (692,719 Wh/m^2^ at 25 m scale), and the lowest topographic wetness index (TWI mean = 5.47 at 25 m scale).

The Meadow site was wet and moderately open with strong midday exposure. While the TWI was moderate (6.58), this site’s solar radiation was the highest (760,684 Wh/m^2^). This site had a mixed vegetation community including grass-shrub covers, evergreen forest edge, and woody wetland cover (25.0%, 33.3%, and 50.0%, respectively).

The Forest Edge site was in the ecotone between the evergreen and deciduous forest (80.0% and 20.0%, respectively) and the mixed vegetation of the adjacent meadow; this site had the lowest solar radiation exposure (186,881 Wh/m^2^) and the highest TWI (7.19).

The key environmental variables were strongly correlated. Evergreen forest cover correlated with elevation at r = −0.996 (*p* < 0.001) and with TWI at r = 0.972 (*p* < 0.001), and soil moisture (TWI) correlated negatively with elevation at r = −0.989 (*p* < 0.001).

### 2.4. Statistical Analysis

All statistical analyses were conducted in R (v4.4.2) [30], using the tidyverse, ggplot2, lme4, glmmTMB, blme, gllvm, and emmeans packages.

We used Pearson correlations to examine pairwise relationships between all primary ecological variables, environmental variables (TWI, solar radiation, elevation), and NLCD vegetation cover classes to detect potential relationships.

We used multiple strategies to test for associations between fly species on flower heads. First, we used Chi-squared analysis to test whether pairs of species were more likely to co-occur on a single flower head than expected. We tested for this association across all flower heads, as well as testing whether co-occurrence rates varied by site. Because the Chi-square analysis did not account for the nested structure of the data, leading to pseudoreplication, we confirmed our conclusions using a generalized linear latent variable model (GLLVM) with plant as a random effect to test for overall co-occurrence patterns. We then used a likelihood ratio test to compare GLLVMs with and without a site effect to test whether co-occurrence patterns varied by site. Finally, we used correlation analyses to test for pairwise relationships between the abundance of the fly species found at the site. For the latter analysis, we tested the correlations using residual fly abundance after using a negative binomial regression to account for the effect of flower head diameter, which had a positive association with fly abundance.

To test whether the abundance of the tephritids in flower heads is affected by individual plant characteristics, we used negative binomial regressions with flower head diameter, phenological head number, and flowering stage as fixed effects and plant as a random effect. We tested the effect of these plant characteristics both on total fly abundance and on the abundance of the individual species found in the flower head. We also tried using site as an additional predictor to test for interactions between site and plant characteristics. However, most models with site did not converge; the ones that did showed no significant interactions, so we removed site as a variable from the final models. Following model fitting, we used Wald Chi-square tests to test the significance of each effect. When the Chi-square tests produced significant results (*p* < 0.05), we used post hoc tests with Tukey’s Honest Significant Difference correction to make pairwise comparisons when necessary.

To test for differences in fly presence, fly abundance, and herbivore damage between habitats, we used generalized linear mixed models (GLMMs) with habitat as a fixed effect and plant as a random effect. We used Wald Chi-square tests and post hoc tests to test the significance, as described above.

Fly presence was modeled using a binomial regression. Fly abundance was measured as counts of flies in each thistle head; since we found these counts to be over-dispersed, we used a negative binomial GLMM to model these data.

To test whether the frequency of tunneling damage, imposed specifically by *Chaetostomella undosa* and so a proxy for its occurrence, varied between habitats, we fit a binomial regression model. Because the high site had 100% frequency of tunneling damage, we used a Bayesian regression, fit with the blme package in R. We included a weakly informative prior (Normal(0, 2.5)) on fixed effect coefficients to prevent coefficients from drifting to infinity.

We calculated the proportion of damaged seeds in each flower head from counts of the total number of seeds and the number of damaged seeds remaining in each head; therefore, we modeled these data using a binomial distribution, with the total number of seeds as the number of trials and the number of damaged seeds as the number of successes.

Stem-receptacle and receptacle surface damage were directly estimated proportions; so, we modeled these data with a beta distribution. However, because there were flower heads with 100% damage, we squeezed the data with a Smithson and Verkuilen transformation, which shifts the proportion of damage to slightly below 1 for heads with 100% damage. We chose this method because, biologically speaking, 100% damage is unlikely to be significantly different from a marginally lower level of damage.

## 3. Results

### 3.1. Tephritid Fly Abundance and Feeding Guild Composition

Tephritid flies were present in 61.8% of the dissected *Cirsium funkiae* flower heads (*n* = 317) across the three habitats at the end of the growing season. Other insects were infrequent in the dissected flower heads (total *n* = 79). The most common of these (81%) were tiny (1–2 mm) vermiform larvae (Diptera), and the most common herbivorous insects (10%) were plant bugs (Miridae: Heteroptera). The other herbivores that we found were rare: only one microlepidopteran moth larva and two very small flower beetles. We also found three individual parasitoids (2 Pteromalidae, 1 unknown microhymenopteran) and one predator (Ladybug beetle). Thus, a few other insects contributed to the feeding guild within the flower heads.

Three tephritid species were identified. *Terellia occidentalis* was the most widespread species (*n* = 327 flies), present in 62.2% of fly-containing heads recorded. *Paracantha gentilis*, which pupates within the flower heads, was next most common (*n* = 258 flies) and present in 52.0% of those heads. Finally, *Chaetostomella undosa* was least common (*n* = 200 total flies), occurring in 43.9% of the heads with flies. Two additional small fly larval types were recorded but are excluded from analyses due to their small sample sizes (*n* = 14 tiny flies, and *n* = 19 unknown flies found on only 14 heads each), and states that prevented them from being identified.

The life stages of flies recovered varied notably among species and sites (Table 1). *Terellia occidentalis* was represented predominantly by pupae across all three sites, consistent with its broad presence and high overall abundance. *Paracantha gentilis* showed a striking difference between sites. Meadow Site yielded almost exclusively larvae (*n* = 133) with virtually no pupae or adults, while Forest Edge was dominated by pupae (*n* = 61) with larvae nearly absent. This shows a potential phenological difference between the sites.

**Figure 3:**
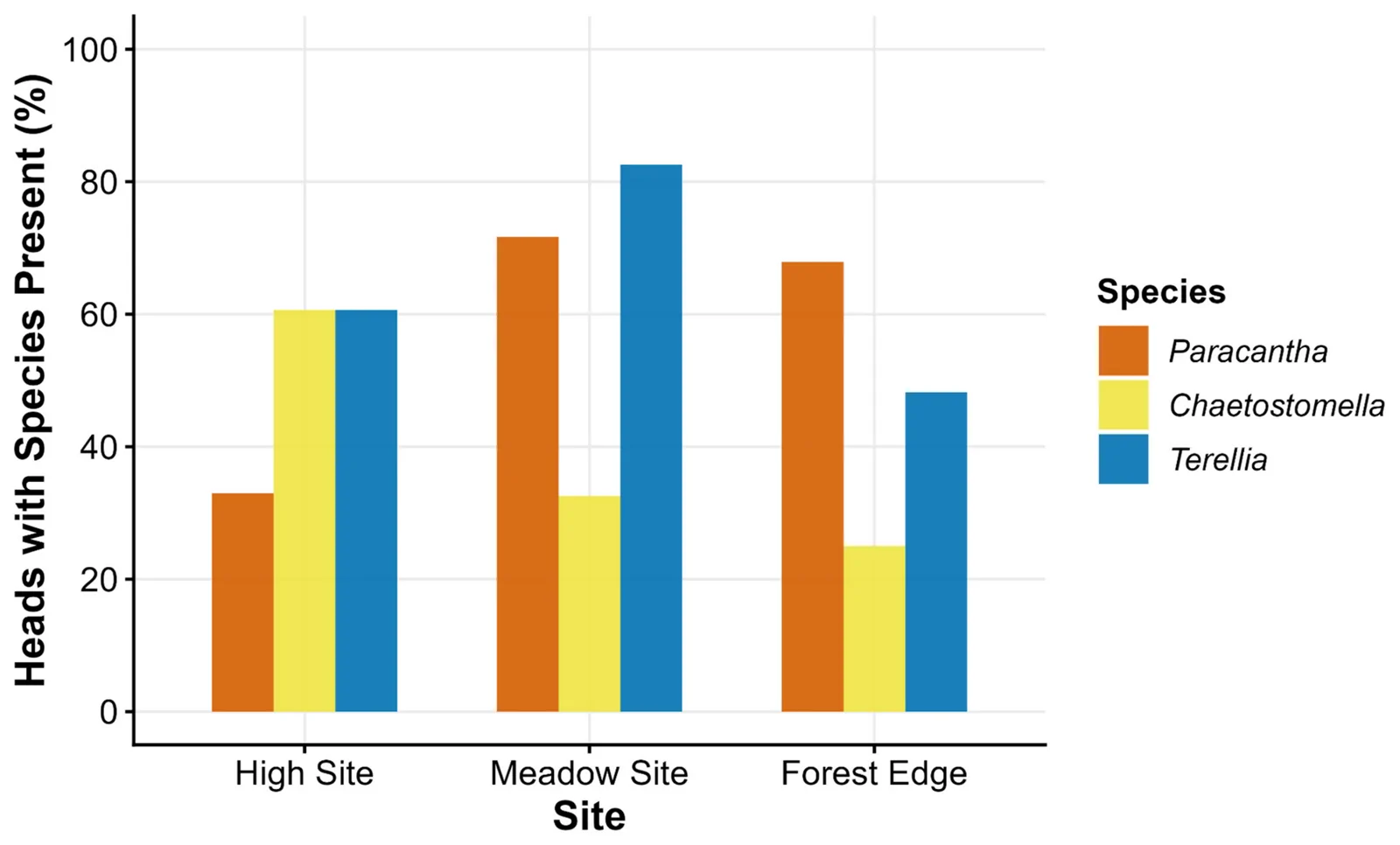
The rate of presence in each habitat for *Paracantha gentilis* (orange), *Chaetostomella undosa* (yellow), and *Terellia occidentalis* (blue), measured as the percentage of heads with the species present.

Presence rates differed substantially among habitats. *Chaetostomella* was predominant in the High site; it was present in 60.6% of High site heads but only in 32.6% and 25.0% of Meadow site and Forest Edge site heads, respectively (Figure 3, yellow bars; *χ*^2^ = 19.07, df = 2, *p* < 0.001). Alternately, *Paracantha* was relatively sparse in the High site, compared to the other two sites; it was present in 71.7% of heads at the Meadow site and 67.9% of heads at the Forest Edge site, but only in 33.0% of heads at the High site (Figure 3, orange bars; *χ*^2^ = 19.64, df = 2, *p* < 0.001). *Terellia* was present at all three sites at relatively high rates, but peaked in the Meadow site on 82.6% of heads with fly larvae, compared to in 60.6% of heads at the High site and only 48.2% of heads at the Forest Edge site (Figure 3, blue bars; *χ*^2^ = 11.3, df = 2, *p* < 0.003).

**Figure 4:**
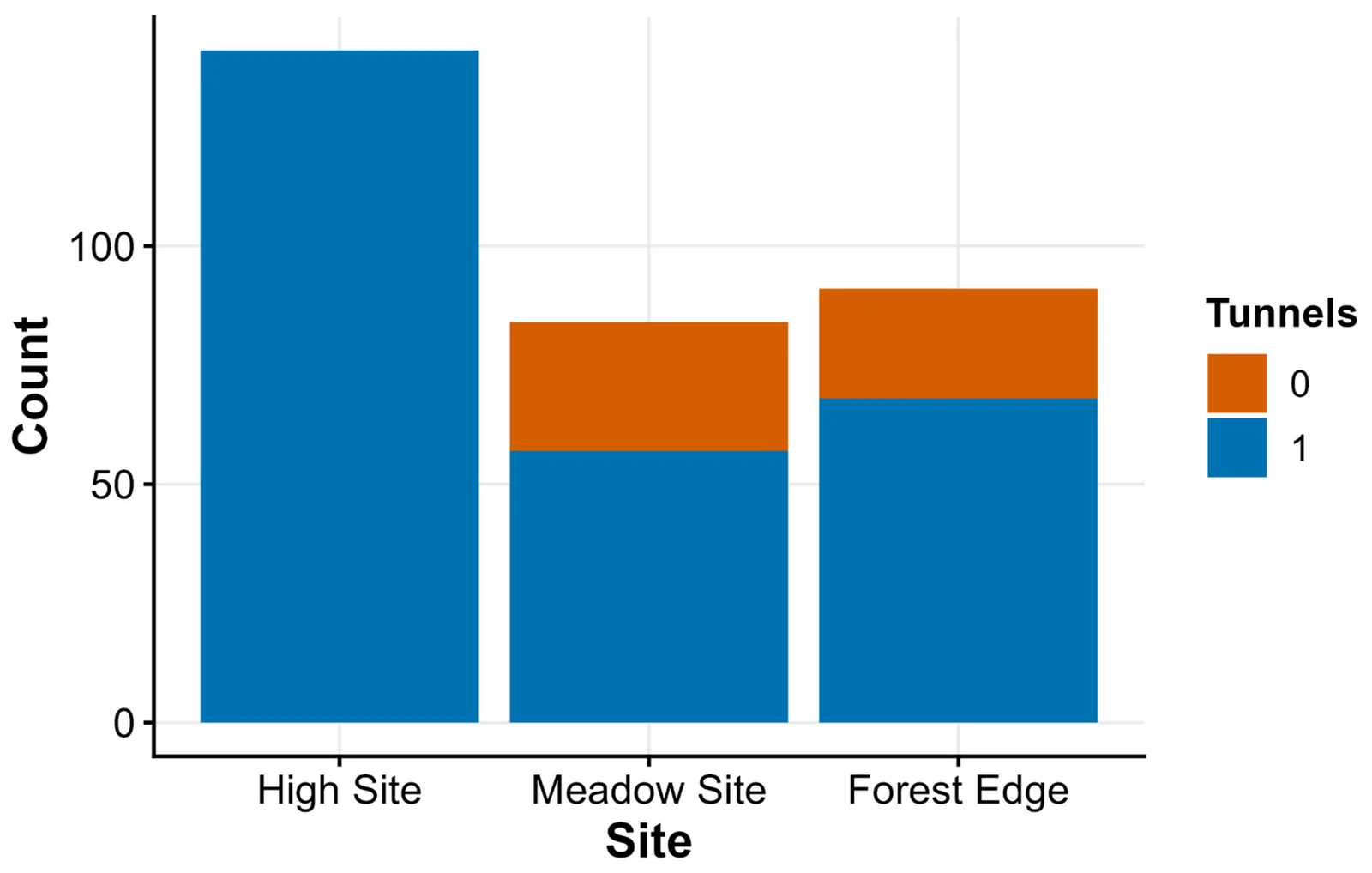
Frequency of tunneling damage by *Chaetostomella undosa* in flower heads of *Cirsium funkiae* by habitat. 0 (orange) = no tunneling damage, 1 (blue) = tunneling damage.

The presence of tunneling damage, done by *Chaetostomella undosa* and so a proxy for its occurrence, also differed significantly between sites (Figure 4, *χ*^2^ = 16.253, df = 2, *p* < 0.001). Every single flower head examined from the High site had tunneling damage. The frequency of tunneling damage at the High site (frequency = 1.000) was significantly higher than that at the Meadow site (frequency = 0.679, *p* = 0.0002) and that at the Forest Edge site (frequency = 0.747, *p* = 0.0005). The frequencies of tunneling damage at the Meadow and Forest Edge sites did not differ from each other (*p* = 0.61).

**Figure 5:**
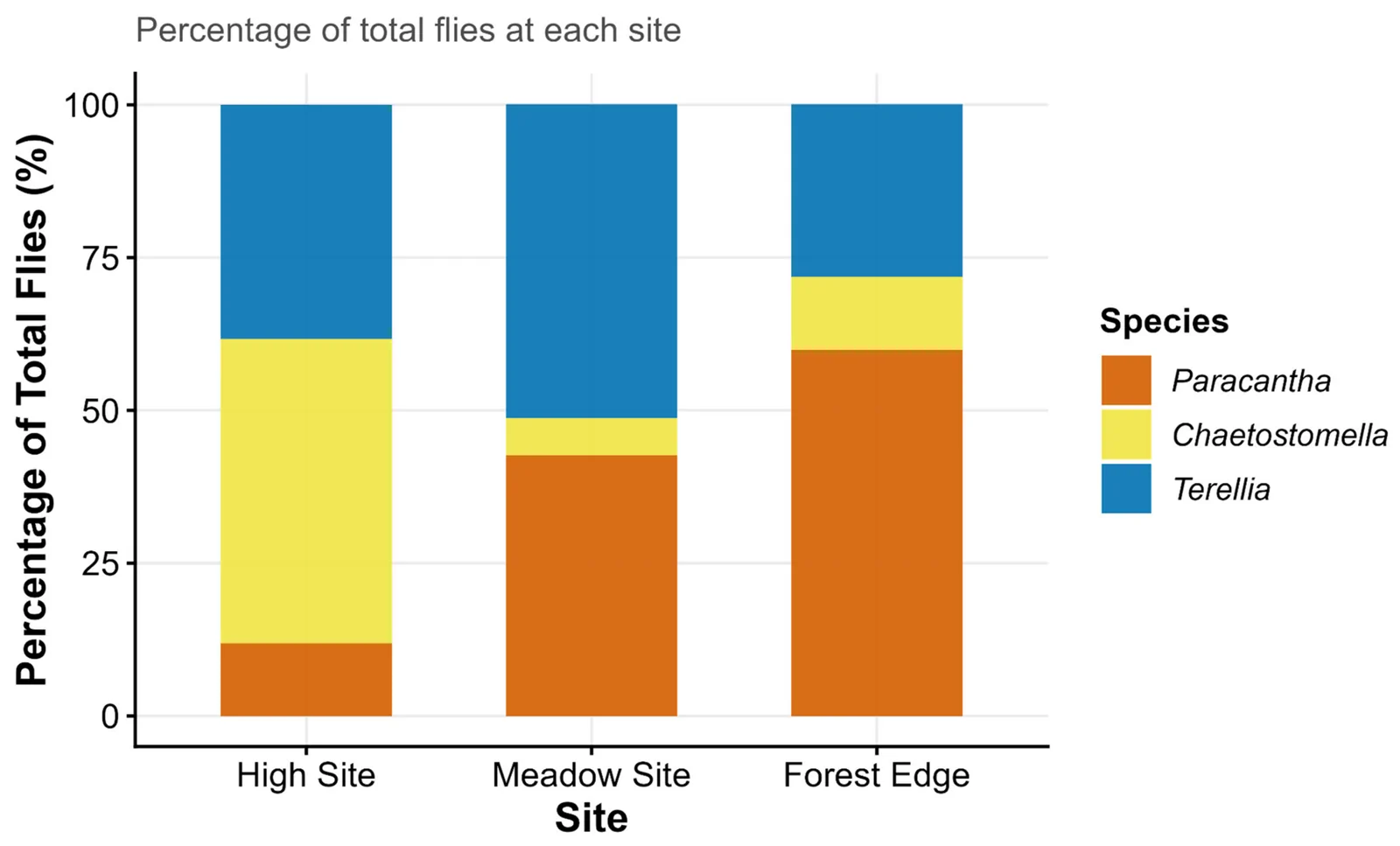
The proportional (%) composition of tephritid fly species as a percentage of total flies recorded at each site (High site *n* = 329, Meadow site *n* = 314, Forest Edge site *n* = 142).

**Figure 6:**
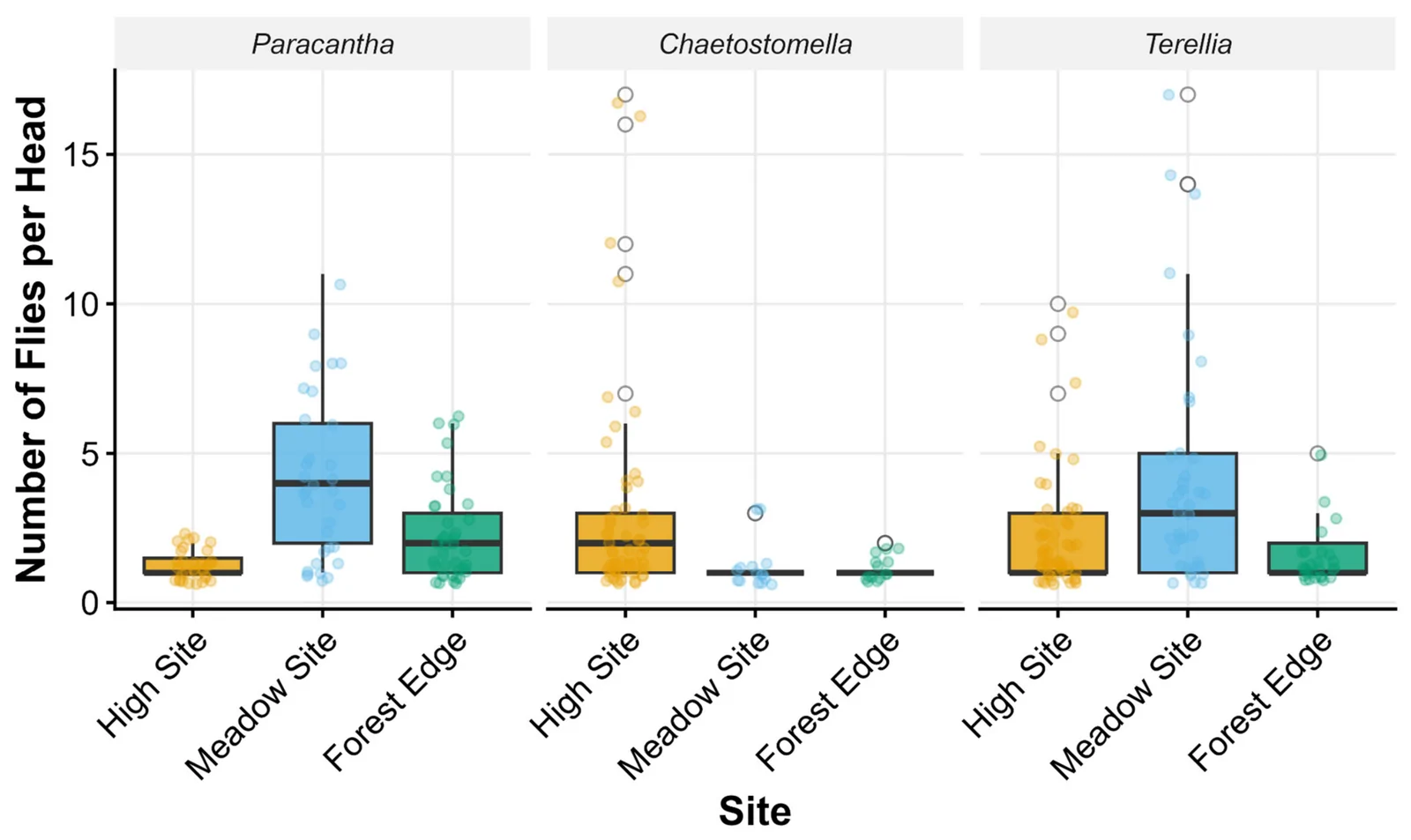
The number of flies observed per flower head at each site for *Paracantha gentilis*, *Chaetostomella undosa*, and *Terellia occidentalis*. Counts include only flower heads in which each species was observed. Colors indicate site: yellow = High Site, blue = Meadow Site, green = Forest Edge. Grey, open circles represent outlier values beyond 1.5 times the inter quartile range.

Tephritid fly species abundance and guild composition differed substantially among the three sites. First, *Chaetostomella* abundance differed significantly across sites (*χ*^2^ = 16.69, df = 2, *p* < 0.001). This species dominated at the High site, where it made up 49.8% of all flies recorded there (Figure 5, yellow bars); it averaged 2.88 flies per head when present (Figure 6, middle panel). However, *Chaetostomella* comprised only 6.1% of the flies observed at the Meadow site and only 12.0% of the flies at the Forest Edge site. Second, while *Paracantha* abundance differed significantly among sites (*χ*^2^ = 26.64, df = 2, *p* < 0.001), it showed the opposite pattern to that of *Chaetostomella*. *Paracantha* dominated at the Forest Edge site (59.9% of flies: Figure 5, orange bars); it averaged 2.24 per head when present there (Figure 6, left panel). In contrast, *Paracantha* represented only 11.9% of the flies at High site and 42.7% at the Meadow site. *Terellia* abundance also differed significantly across sites (*χ*^2^ = 18.27, df = 2, *p* < 0.001), even though it was present at high relative abundance in all three sites (28.2–51.3%; Figure 5, blue bars). *Terellia* numbers peaked at the Meadow site, where it averaged 4.24 flies per head (Figure 6, right panel). The significant variation among sites for all three species reflects differences in abundance across the three sites; these differences were confirmed by pairwise contrasts.

However, when plant was included as a random effect in the model, total fly abundance was only marginally associated with site (*χ*^2^ = 4.83, df = 2, *p* = 0.089), with no significant pairwise contrasts between sites. This means that, while the composition of the fly guild differed with habitat, the total number of flies on any given flower head was determined more strongly by the individual plant traits, like average head diameter, than by the site at which the plant was found. Thus, the inference is that large flower heads at the High site attracted as many flies as large flower heads at the Meadow site, even though the fly species differed (see below).

### 3.2. Tephritid Species Co-Occurrence

The species combinations within individual heads reflected the differences in fly species abundance and occurrence documented above, though sample sizes for individual combinations were modest. For example, *Chaetostomella*, the predominant fly at the High site, was found alone most frequently there (*n* = 23 of 94 heads). *Paracantha* and *Terellia* were found together most commonly at the Meadow site (*n* = 20 of 46 heads). Additionally, *Paracantha*, the predominant fly at the Forest Edge site, was found alone most frequently there (*n* = 17 of 56 heads).

Further, we found that no species pair had a significant association representing co-occurrence across all flower heads. However, the patterns of co-occurrence between species did significantly differ by site (GLLVM: D = 64.86, df = 6, *p* < 0.001). This was further supported by the Chi-square analyses comparing co-occurrence for pairs of species across sites, showing that the conclusions are robust to analysis decisions. *Paracantha* and *Terellia* co-occurred on 63.0% of heads at Meadow site, compared to 22.3% at High site and 28.6% at Forest Edge site, a difference that was highly significant, as confirmed by both the Chi-square analysis (*χ*^2^ = 23.82, df = 2, *p* < 0.001). There was a marginally significant difference in co-occurrence of *Chaetostomella* and *Terellia* across sites (*χ*^2^ = 4.64, df = 2, *p* = 0.098), with the highest co-occurrence at the High site (28%) and the lowest at the Forest Edge site (8%). Co-occurrence rates did not vary significantly by site for *Chaetostomella* and *Paracantha* (*χ*^2^ = 0.25, df = 2, *p* = 0.88).

**Figure 7:**
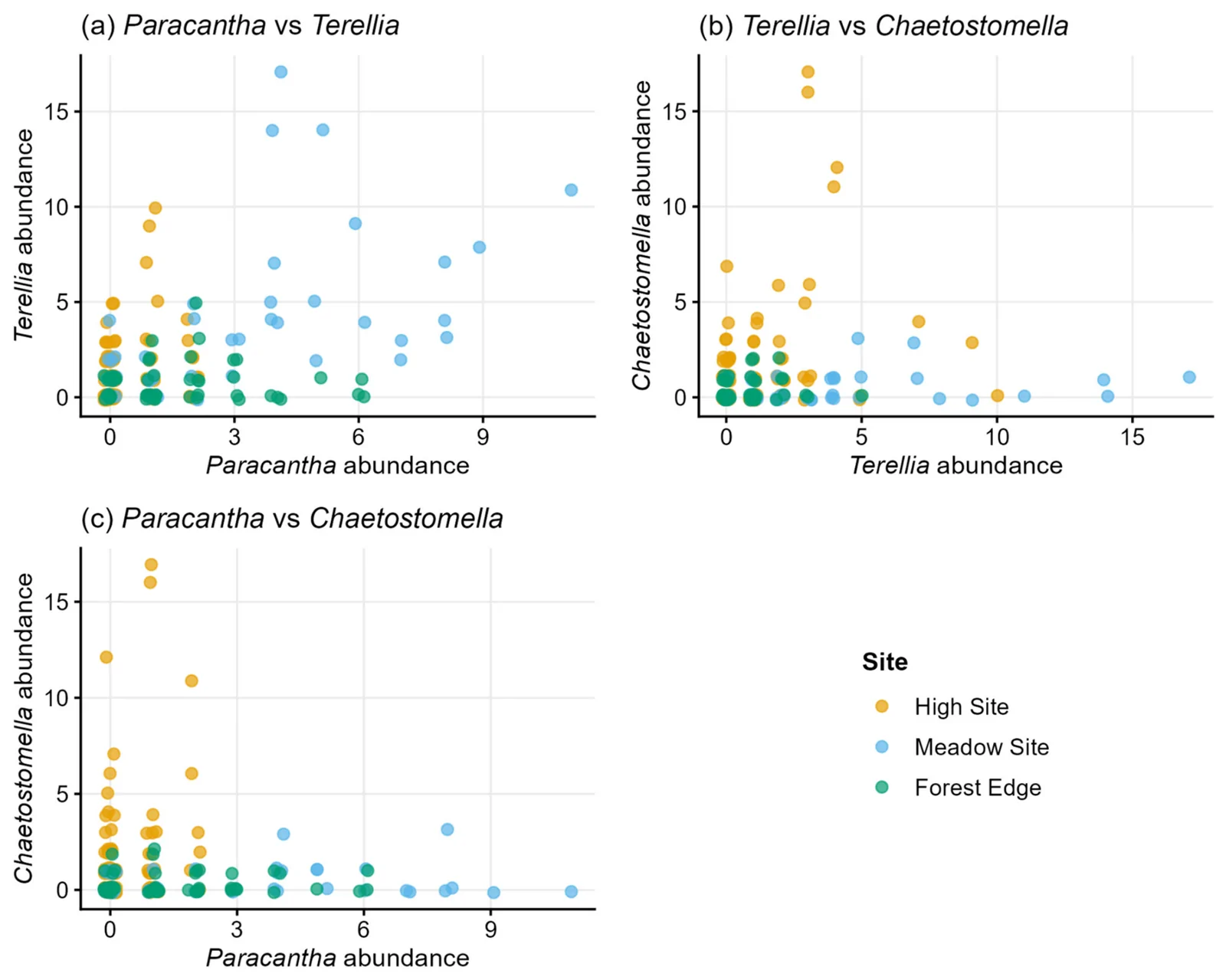
The relationship between the abundance of pairs of tephritid fly species observed on *Cirsium funkiae* flower heads for (**a**) *Paracantha gentilis* and *Terellia occidentalis*, (**b**) *Terellia occidentalis* and *Chaetostomella undosa*, and (**c**) *Paracantha gentilis* and *Chaetostomella undosa*. Points are colored by site: yellow = high site, blue = meadow site, and green = forest edge site.

Flower head diameter was a significant positive predictor of abundance for both *Paracantha* and *Terellia*. Numbers of both fly species increased significantly with an increase in flower head diameter. After the effect of head diameter on fly abundance was removed, there was still a positive relationship between *Paracantha* and *Terellia* abundances on the same heads (Figure 7a; r = 0.27, df = 194, *p* < 0.001). Thus, head diameter influenced the association between the two species, but it was not the only parameter driving it. This result is related to the specific fly abundances at the Meadow site (Figure 7a, blue points) itself, since that site had higher overall abundances of both species.

*Chaetostomella* abundance did not vary significantly with the abundance of either *Terellia* (Figure 7b; r = 0.044, df = 194, *p* = 0.541) or that of *Paracantha* (Figure 7c; r = −0.07, df = 194, *p* = 0.33). Thus, *Chaetostomella* occurrence was independent of the other two tephritid species, most likely related to the observed differences in feeding niche, stems, and receptacle tissues versus flower head ovules and seeds.

### 3.3. Influence of Plant Variation on Fly Occurrence

**Figure 8:**
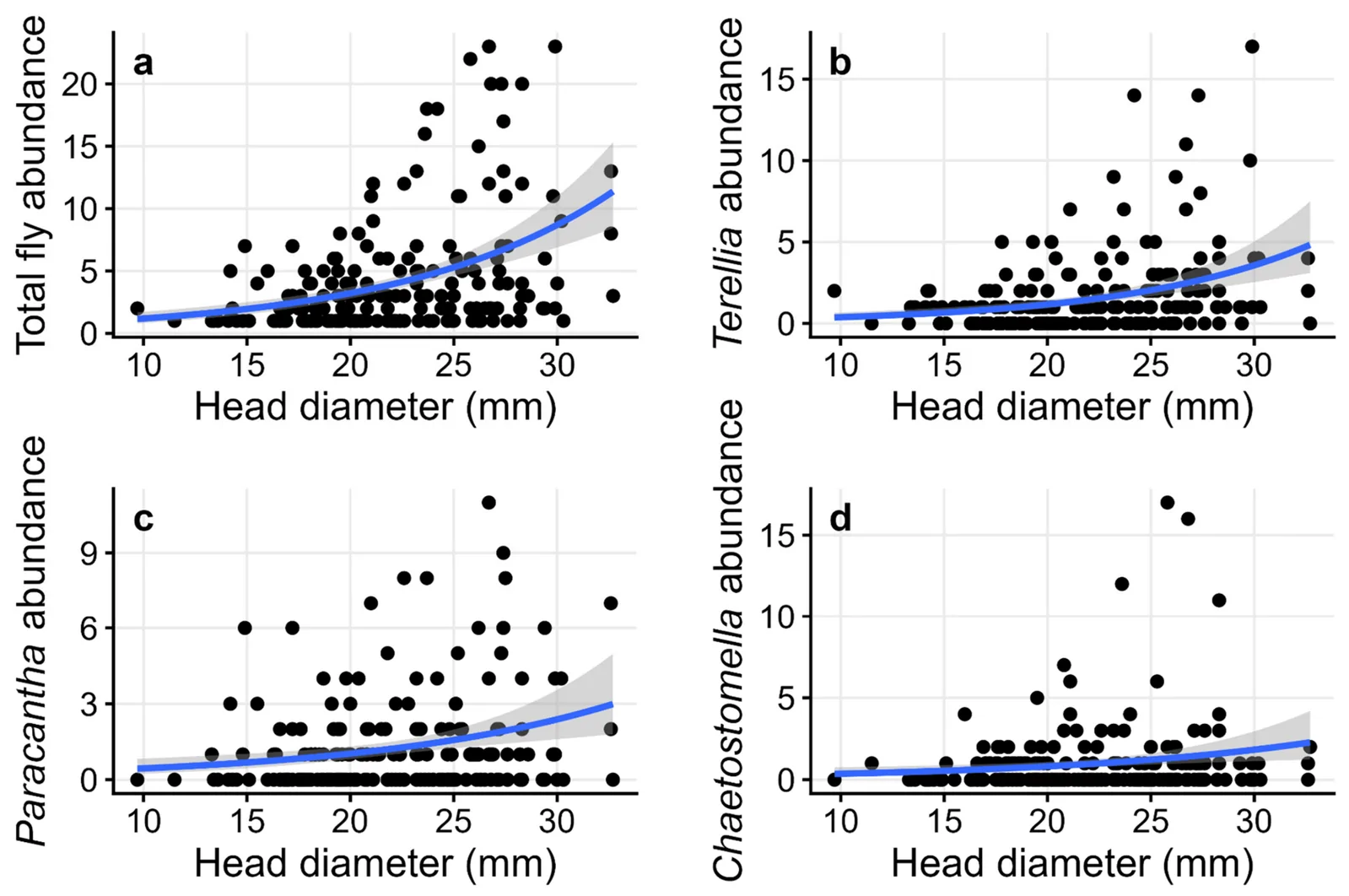
The relationship between *Cirsium funkiae* flower head diameter and the (**a**) total fly abundance, (**b**) *Terellia occidentalis* abundance, (**c**) *Paracantha gentilis* abundance, and (**d**) *Chaetostomella undosa* abundance in flower heads. Each point represents an individual flower head. The blue line indicates the fitted regression line and the grey shading represents the 95% confidence interval.

Flower head diameter was a significant positive predictor of total fly abundance within flower heads (Figure 8a; *χ*^2^ = 23.46, df = 1, *p* < 0.001). This result was driven largely by a strong positive relationship between head diameter and *Terellia* abundance (Figure 8b; *χ*^2^ = 19.76, df = 1, *p* < 0.001). However, there were also marginally significant effects of head diameter on abundance of both *Paracantha* (Figure 8c; *χ*^2^ = 2.8, df = 1, *p* = 0.094) and of *Chaetostomella* (Figure 8d; *χ*^2^ = 3.4, df = 1, *p* = 0.065). Flower head diameter did not differ significantly across sites (F = 0.189, *p* = 0.828), confirming that site-level differences in fly abundance and damage were independent of any site differences in flower head size.

Flowering stage was also a significant predictor of the abundance of *Paracantha* (*χ*^2^ = 7.45, df = 2, *p* = 0.024), and marginally so for *Terellia* abundance (*χ*^2^ = 4.82, df = 2, *p* = 0.09). Flowering stage was not significant in *Chaetostomella* abundance (*χ*^2^ = 0.91, df = 2, *p* = 0.63), nor for total fly abundance (*χ*^2^ = 4.51, df = 2, *p* = 0.1). *Paracantha* was more abundant in flower heads in the dispersing stage than in the flowering stage (*p* = 0.046), while *Terellia* was marginally more abundant in the bud stage than the flowering stage (*p* = 0.074). No other pairwise comparisons were significant. The distribution of flower head stages differed significantly across sites (*χ*^2^ = 17.042, df = 4, *p* = 0.002); the flower heads at the High site were predominantly in the flowering stage (89.5% of heads), while those at Forest Edge and Meadow Site were proportionally more in the bud and dispersing stages (31.4% and 37.3% combined, respectively).

Phenological head number did not have a significant effect on either total fly abundance or the abundance of any individual fly species, either overall or when compared at the site-level.

Overall, the floral characteristics we tested explained more variance in both total fly abundance (marginal r^2^ = 0.227) and in *Terellia* abundance (marginal r^2^ = 0.215) than it did in either *Paracantha* abundance (marginal r^2^ = 0.068) or *Chaetostomella* abundance (marginal r^2^ = 0.062). This pattern did not vary among habitats.

### 3.4. Intensity of Herbivory and Seed Reproductive Success

A multiple regression analysis that included individual fly species counts, flower head diameter, receptacle surface damage, and stem-receptacle damage explained 24.7% of variance in the proportion of seed damaged (Adj R^2^ = 0.223, *p* < 0.001). The stem-receptacle damage contributed significantly (β = 0.051, *p* = 0.009); since this damage represents feeding by *Chaetostomella*, this result suggests increased abundance of this fly led to higher seed damage proportions. Unexpectedly, *Paracantha* abundance showed the opposite relationship. Heads with more *Paracantha* flies tended to have somewhat lower seed damage proportions after accounting for the other variables (β = −0.040, *p* = 0.034). Variation in *Terellia* abundance did not contribute independently to variation in seed damage proportion after the other variables were included in the model (*p* = 0.306). In sum, the three tephritid species differ not only in their habitat preferences and flower-head selection, but also in their subsequent associations with seed damage. These results suggest that *Chaetostomella* increased the damage level, while *Paracantha* was associated with somewhat lower damage, and *Terellia* showed no independent effect once the other species are accounted for.

**Figure 9:**
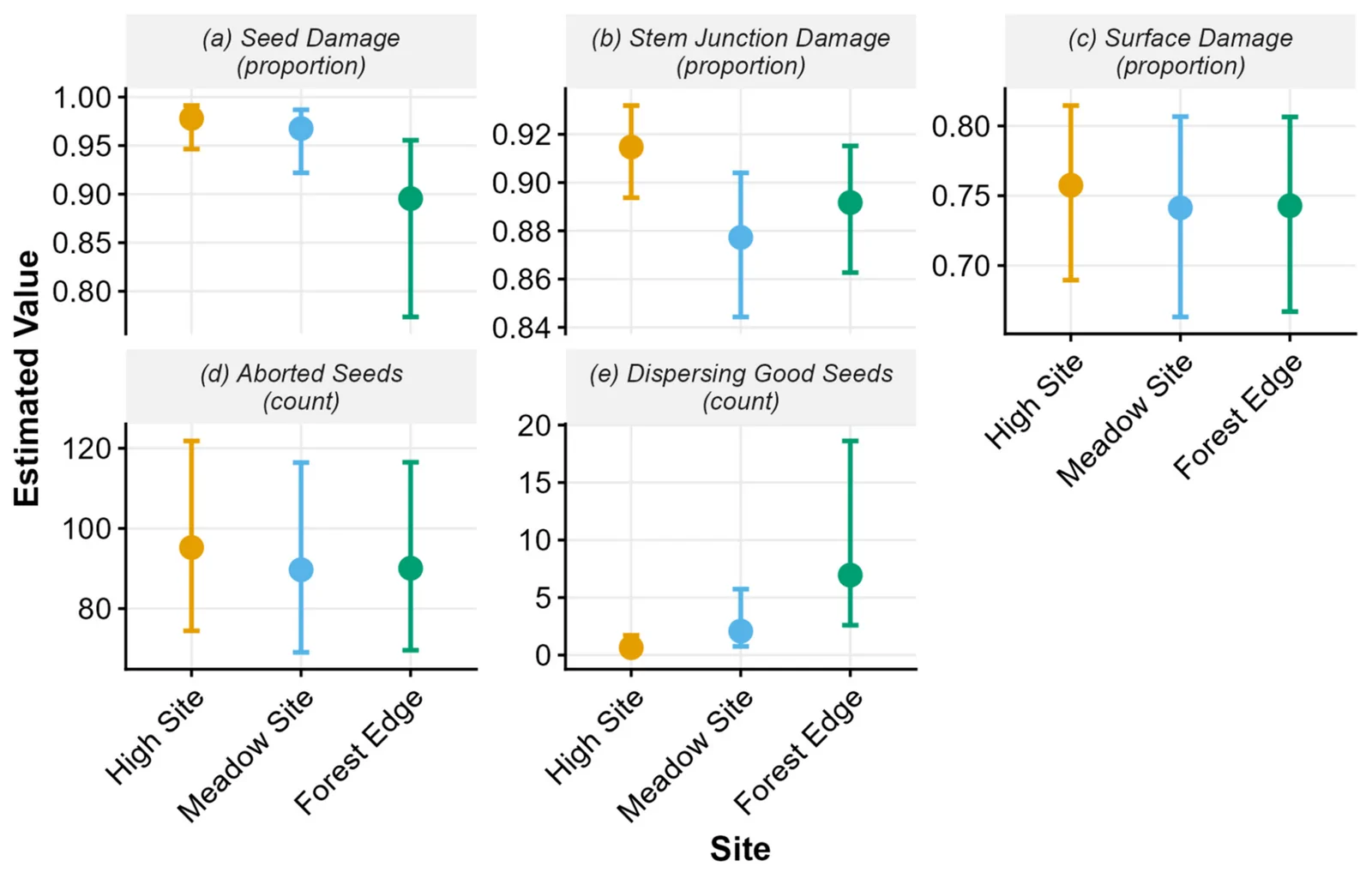
Variation by site in the (**a**) proportion of seed damaged, (**b**) proportion of stem-receptacle junction damaged, (**c**) proportion of receptacle surface damaged, (**d**) number of aborted seeds, and (**e**) estimated number of “good” potentially viable filled and undamaged seeds per *Cirsium funkiae* flower head. Yellow = high site, blue = meadow site, and green = forest edge site.

The tephritid feeding guild imposed very high levels of pre-dispersal seed predation and loss across all sites: 98.5%, 95.8%, and 91.5% at the High site, Meadow site, and Forest Edge site, respectively (Figure 9a). Despite this relatively uniform high damage, the site differences were actually statistically significant (binomial GLMM: *χ*^2^ = 6.69, df = 2, *p* = 0.035). The Forest Edge site flower heads had significantly lower seed damage than did those at the High site (*p* = 0.035).

Damage to the whole receptacle base, the stem junction-receptacle here reflected the amount of direct feeding on the main stem, the subtending flower head stem, and the receptacle tissues underlying the receptacle surface by *Chaetostomella*. This stem junction-receptacle feeding damage differed significantly across sites (Figure 9b; beta GLMM: *χ*^2^ = 8.31, df = 2, *p* = 0.016). The flower heads at the High site experienced significantly greater stem junction-receptacle damage than did those at the Meadow site (*p* = 0.017). However, receptacle surface damage, representing primarily feeding by *Paracantha* and *Terellia*, did not differ significantly among sites (Figure 9c; *χ*^2^ = 0.149, df = 2, *p* = 0.928). Also, the number of aborted seeds per flower head did not differ among sites (Figure 9d; *χ*^2^ = 0.137, df = 2, *p* = 0.934).

The clearest effect of the site-level differences in feeding by the tephritid fly guild above showed up in realized viable seed production (Figure 9e). Unexpectedly, flower heads at the Forest Edge site produced and dispersed significantly more good, putative viable seeds than did those in the more typical habitat of the High site (negative binomial GLMM: *χ*^2^ = 11.27, df = 2, *p* = 0.004; pairwise contrast *p* = 0.002). This was the strongest, most consistent site difference in flower-head seed production within the dataset.

The proportion of viable seeds per flower head estimated in this study showed the same pattern. Heads from the plants at the Forest Edge site had the highest proportion of estimated viable seeds (8.2%), compared to both those at the Meadow Site (5.1%) and at the High Site (1.6%).

Also, interestingly, seed damage was negatively associated with flower head diameter (R^2^ = 0.044, *p* = 0.003), despite the finding that fly abundance was positively associated with flower head diameter. This may reflect the fact that early feeding damage during development caused flower heads to abort in the bud and early flowering stages of flower-head development.

## 4. Discussions

### 4.1. Fly Abundance and Guild Composition with Site and Habitat

One of the more unexpected results of this study was the clear dominance of internal-feeding tephritid fly pre-dispersal seed predators as the primary herbivores of *Cirsium funkiae* across all three high alpine sites. *Cirsium* spp. thistles in other ecosystems typically have a diverse guild of herbivorous insects, including aphids, treehoppers, leaf beetles, weevils, and various moth larvae [31,32]. However, we did not observe that variety in this system. This outcome likely reflects the high, treeline, or above elevation of this thistle species. Harsh high alpine conditions can limit activity and survival of external-feeding, ectothermic organisms (see, e.g., [33]). In addition, these thistles have physical defenses, such as strong hairiness and sharp spines, and they occur in an environment with short, harsh growing seasons that limit vegetative productivity. Both plant defenses and harsh growing conditions likely contribute to limiting the range of herbivores capable of utilizing these thistles as host plant resources.

The abundances of the three tephritid species varied significantly among the three sites. Composition also varied among the sites, but not as much. *Chaetostomella undosa* was dominant in the highest, most exposed talus habitat (High site), typical of the plant, with evidence of its occurrence and feeding in 100% of flower heads examined. *Paracantha gentilis*, a species recorded occurring on other *Cirsium* spp. in the adjacent foothills such as on *C. canescens*, was most abundant in the lowest site, Forest Edge; this suggests the fly’s range extends up from lower elevations. However, the Forest Edge site was also the most sheltered site and had plants growing well on the damp soils of the forest edge—meadow ecotone. Finally, *Terellia occidentalis*, a foothills thistle species [21], had relatively high abundances across all three sites, suggesting a broad range; however, its numbers peaked in flower heads on plants at the Meadow site, in a semi-exposed location on moist soils at the intermediate elevation. This suggests that *Terellia* performance decreases at the highest, most exposed location typical of *C. funkiae*, at least relatively. Future studies should replicate habitats and elevations to evaluate the generality of these findings and further partition habitat vs. elevation and site influence.

Similar variation in abundances among overlapping tephritid species on a single host plant species has been documented in lower elevation *Cirsium* systems in Europe [34], in the southwestern USA [35], and in this region in the foothills of the central Rocky Mountains, USA [21]. However, none of the previous studies of the palearctic genus *Cirsium* have examined high alpine species. Also, we found only two studies of tephritids on alpine Asteraceae that have been done globally, one in New Zealand [36] and one in Australian alpine [10]. Those studies were extensive, examining a small number of flower heads per species for a large number of species of alpine Asteraceae. This study instead was intensive; we examined a large number of flower heads for one species of thistle, *Cirsium funkiae* (Asteraceae), in three high alpine sites. Interestingly, our results are consistent with a subset of alpine Asteraceae species that experienced high frequencies of tephritid fly occurrence (5 out of 23 species examined) in Australia [10]. In particular, both Pickering [10] and we found that flower head size (diameter) was an important parameter in the interactions, at least for two of the three species we observed.

Environmental differences between the three sites could help explain the variation in tephritid fly abundances observed, either directly or indirectly in their influence on the plants. The High site was characterized by dry rocky soils and had exposure to high solar radiation. The Meadow site, along Crestone Creek at the intermediate elevation, had wet soil and semi-exposed conditions with high solar radiation only in the middle of the day. The Forest Edge site, at the lowest elevation, had moist soils influenced by the creek but further from it and the least exposure to solar radiation, just at midday. While strong intercorrelation among the environmental variables (exposure, elevation, soil moisture) at each site prevents the identification of a specific environmental characteristic as a driver in this study, these environmental differences, and associated differences in vegetation structure, can influence fly abundance and guild composition, both directly and indirectly. Effects could include environmental influences on adult fly flight and oviposition or on host plant quality, phenology, or defenses. Future studies with replicate sites for habitats and elevations are needed.

*Chaetostomella*’s dominance at the harshest, most exposed site may reflect a thermal advantage in open talus, where surface temperatures can exceed air temperatures and create microenvironments that favor certain insects [13]. Temperature has been shown to positively influence tephritid oviposition behavior at the individual plant level, while humidity influences it negatively [37], suggesting that the dry, thermally extreme conditions at High Site may actively favor *Chaetostomella* oviposition over the other two species. Further studies could alter growing conditions experimentally, as well as extend sampling to sites at the same elevation that varied in soil moisture and/or exposure, to partition influences definitively.

Also, the flower head samples were collected at the end of the growing season, specifically to allow assessment of the consequences of the interactions for plant reproductive success. Thus, the fly counts represent only those flies present then, rather than throughout the growing season. The counts for *Paracantha gentilis* are a good estimate of the number of successful individuals produced since this species pupates within the flower head. Both *Chaetostomella undosa* and *Terellia occidentalis*, however, tend to drop out of the flower head to pupate elsewhere. However, we were able to approach the temporal issue for *Chaetostomella* by analyzing the tunneling data that reflected its feeding. Further, Pickering [10] on the Australian Asteraceae found that flowering time did not significantly impact assessment of tephritid occurrence; these suggest that our end-of-season collection, required to document seed production, provided a reasonable estimate of relative abundance. Future studies could improve our understanding of tephritid abundance and guild composition in relation to environmental variation by examining numbers in relation to the phenological development of the flower heads, and by discovering pupation sites for *Chaetostomella* and *Terellia*.

### 4.2. Co-Occurrence of Tephritid Species in Relation to Site Variation

The notable co-occurrence of *Paracantha* and *Terellia* at the intermediate Meadow site, where the two species shared heads at more than twice the rate observed at the other two sites, suggests that the wet meadow habitat provided conditions that allow resource sharing. While the counts for individual species combinations were modest and should be interpreted with caution pending site replication and larger sample sizes, this pattern of high co-occurrence persisted after removing the effect of head size (diameter); this confirms that the elevated co-occurrence rate is not simply a species preference for larger heads, or a resource concentration effect. One possible hypothesis to explain high co-occurrence of the two species in the Meadow site is that the open, riparian edge conditions support plant growth and higher resource quality and availability, allowing multiple floral herbivore species to overlap simultaneously. The Meadow site may also represent an area of enhanced range overlap, meeting environmental preferences of both species, resulting in higher co-occurrence. Since there was no significant overall association between the two species, *Paracantha* and *Terellia* do not necessarily avoid or associate, but co-habitate when certain conditions are met. Added sampling of similar habitat sites at the same elevation in future studies would improve understanding and predictability of this pattern of co-occurrence.

*Chaetostomella*, the dominant species in the High site open habitat that is typical of this alpine thistle, showed no significant association with either of the other two tephritid species. We recorded feeding damage by *Chaetostomella* on flowering stems, both the main stem and the subtending flower head stem, as well as in receptacle tissues of flower heads, with heavy mining and chewing damage both low and high on the main stem of the flowering mass. The observations show that *Chaetostomella* was highly destructive on *Cirsium funkiae* stems and developing flower head tissues in its typical high, open talus habitat. We hypothesize that the mature larvae exiting flower heads likely overwinter among the lower leaves and inside the lower main stem tissue, similar to its behavior on *C. cymosum* [22]. In sum, the evidence suggests that *Chaetostomella* selects host plants independent of oviposition by overlapping tephritid species. Future studies using larger sample sizes could confirm this.

### 4.3. Plant Traits in Tephritid Abundances in Relation to Habitat

Flower head diameter at the end of the growing season emerged as an important predictor of tephritid fly occurrence for two of the three species, and thus of the late-season estimate of successful development of these tephritids on *C. funkiae* in the high alpine. Flowering stage reached also contributed to predicting *Paracantha* and *Terellia* abundances, but final flower head diameter remained the strongest and most consistent predictor of fly abundance across all three species and all three sites. Notably, average flower head diameter did not differ significantly across the three sites, meaning that the species-specific relationships with head size documented here reflect genuine differences in insect-hostplant interactions among the three tephritid species, rather than site-specific responses.

Finding a relationship between flower head size reached and the number of tephritid flies in the flower heads is understandable, and it is consistent with the findings of Pickering [10]. She found a positive relationship between flower head diameter and tephritid (*Tephritis* spp.) abundance across 23 species of alpine Asteraceae in New Zealand. Our study adds the first data on a similar relationship between successful fly development and flower head size, but rather within-species across sites for an alpine species of thistle (*Cirsium funkiae*, Asteraceae) in the Rocky Mountains, USA. There are several hypotheses to explore to explain this result and the variation in the strength of the relationship across species. One intriguing possibility is that this relationship is determined by female oviposition preference.

For *Terellia occidentalis*, females oviposit in the marginal florets of either larger flower head buds or newly opened flower heads ([17], S. Louda, personal observation), so it is not surprising that oviposition choice could be affected by and related to flower head size, as represented by flower head diameter.

However, for *Paracantha gentilis*, known to oviposit into small, unopened flower head buds [19,20], female oviposition choice cannot explain the weakly positive relationship of number of flies with flower head size. Instead, we hypothesize that small flower head buds that receive high *Paracantha* eggloads die and abort, terminating fly egg and early larval development and removing those flies, while stopping further flower head development, and larger buds and buds that receive fewer eggs go on to develop and support the development of the successful *Paracantha* pupae recorded at season end, with larger buds more likely to survive to support larval development. This hypothesis is consistent with what we know about *P. gentilis* development [19]. It could be evaluated by further detailed study and experimental protection of small flower head buds over the growing season.

The weak relationship between final head diameter and *Chaetostomella* abundance is consistent with observed distribution of *Chaetostomella* larvae within a plant (Louda et al., unpublished data). Based on the position of the feeding damage and the larvae found, most oviposition was not into flower heads but under flower heads, into the angle between the short flower head stem and the main flowering stem. The larvae observed were either inside the main flower stem or embedded and chewing under and among the compacted mass of flower heads in the terminal flower head aggregation, as well as within individual flower head stems and receptacles, where they left feeding tunnels. Thus, it is not surprising that flower-head size attained was not an important predictor of host resource availability for *Chaetostomella undosa*.

The significant difference in the distribution of flowering stages attained by season end among sites adds a further layer of complexity to the host utilization picture. Flower heads at the High site were predominantly stopped in the flowering stage by the end of the season, whereas those at the other two sites—Forest Edge and Meadow—had proportionally more heads that died in the bud stage and proportionally more reached the dispersing stage. This observation suggests either that the sites differed in phenology of flowering, since all samples were collected on the same date, or, more likely, that the interactions with the flies varied over the season and led to different distributions of flower head stages by the end of the season, or possibly both occurred. Further studies of the phenology of flowering and interaction are merited.

Intriguingly, for *Chaetostomella*, the dominant species at the High typical habitat site, the high frequency of flower heads stopped in development in the flowering stage, and so observed at that stage at the end of the season, suggests a “race” between the developing larvae and the developing seed in flower heads. Destruction of the receptacle tissues and interference with phloem transport were not observed to cause immediate flower head abortion, but cumulative fly feeding damage eventually led to the end of further head and seed development, stopping heads in the flowering stage. For *Paracantha*, oviposition preference for small flower heads could lead to feeding damage that causes flower head buds to abort, stopping both flower head and larval fly development, leaving more buds behind and potentially explaining higher *Paracantha* abundance at season end in the larger dispersing heads, those that did not abort early. For *Terellia*, known as a species that can have a second generation and so high abundances in bud-stage heads at season end, an alternative hypothesis is that the insects observed represent a second generation, explaining its high late-season occurrence, especially in flower head buds. Repeated sampling and documenting phenology of flowering and of damage accumulation across the growing season are needed to fully evaluate these hypotheses.

Once plant variation was accounted for statistically, total fly abundance still varied among sites, but only marginally. This suggests that individual plant characteristics play an important role along with habitat preferences in host utilization and subsequent fly success, at least for two of the three species (*Paracantha*, *Terellia*).

### 4.4. Intensity of Herbivory and Seed Reproductive Success in Relation to Habitat

All three tephritid species together were significant as pre-dispersal seed predators, with seed damage exceeding 90% at all three sites. This finding is consistent with other studies of floral and pre-dispersal seed feeding on large-flowered *Cirsium* spp. in other ecosystems; in those studies, tephritid flies along with other floral- and seed-feeding insect herbivores often collectively destroy the majority of seeds produced in a given season [4,20,37,38,39,40]. However, the recorded magnitude of damage and seed reduction per flower head in this study was even higher than in most other studies.

Interestingly, despite the high levels of damage across all three sites, site-level differences in seed damage were significant. Flower heads at the lower Forest Edge site had significantly less damage, and significantly more viable seeds, than did flower heads in the plant’s characteristic habitat at the exposed talus High site. This suggests that forest conditions could buffer individual plant fitness against insect herbivory, consistent with findings in Nepal [11], and merits further investigation. This result also suggests that the tephritid fly guild composition can make a difference for plant seed reproduction even when damage levels are so high. Supporting this inference, in the multiple regression we found that *Chaetostomella* was closely associated with flower heads that sustained the highest levels of damage, whereas *Paracantha* was more closely associated with flower heads that sustained less damage.

An association between high numbers of *Chaetostomella* larvae and evidence of high levels of feeding damage is logical—this tephritid fly feeds by burrowing in the flowering stem, in and on the subtending stem of a flower head, and in the capitulum receptacle tissues; this feeding inflicts severe damage to the developing support tissues as well as to the florets and seeds, likely cutting off phloem to developing seeds. We hypothesize that development over the season represents a “race to maturation” between the developing seeds and the developing larvae. What factors determine the outcome are currently unknown and merit further research.

More puzzling is the association of lower damage with higher numbers of *Paracantha*. We hypothesize that reduced levels of damage to stems and receptacle base tissues by *Chaetostomella* feeding allow increased successful maturation of *Paracantha*, whose larvae feed on the developing ovules and seeds rather than in the receptacle. Receptacle surface damage, by comparison, showed no significant variation across sites, suggesting that high levels of receptacle surface feeding occur regardless of habitat, guild composition, or fly species. Further work is merited to detect the interactive dynamics of these insect—insect interactions.

Stem and receptacle junction damage, imposed by *Chaetostomella* larval feeding, differed significantly across sites, with heads at the High site showing greater damage, significantly more than those at the Meadow site. This, combined with the dominance of observed *Chaetostomella* larvae in the counts at that site, indicates that this species is the main predispersal seed predator in the typical habitat in the exposed, high, talus slopes, where *C. funkiae* is one of the main plant species in a very sparse vegetation. Further study is needed to understand the fitness and population consequences of the magnitude of this severe reduction in seed reproductive success within the plant’s typical habitat. There is clearly strong selection pressure, likely for smaller flower heads (represented by smaller flower head diameters), given the results here showing the increase in fly abundance related to an increase in flower head size. Our results suggest that guild composition rather than fly abundance per se drives the site-level intensity of herbivory.

The negative association between flower head diameter and feeding damage, despite larger heads harboring more flies, suggests a complex set of interactions that lead to a dilution effect. Larger heads can often contain more seeds, which can result in the appearance of smaller proportions of seeds lost to herbivory despite higher fly counts. While this was not the strongest relationship observed, it is worth examining this possibility with larger sample sizes from more sites as well as experimental evaluation of the individual, population, and metapopulation consequences for *C. funkiae* of seed losses of the magnitude reported here.

The difference in seed output between sites was significant despite high levels of herbivory at all sites. Flower heads at the lower Forest Edge site produced more viable seeds than did plants at the High site in the typical open talus habitat. The viable seed estimates, 8.2% for flower heads at the Forest Edge site compared to 1.6% for heads at the High site, while low in all sites, suggest that microhabitat or site environment had a meaningful influence on successful reproductive output in this study. Further research is merited since this study represents one season, and since seeds in heads already at the seed-dispersing stage at the time of collection could not be fully counted. However, the consistency of the pattern across multiple damage variables and seed output measures indicates that the interactions of this high alpine thistle with the tephritid fly floral-feeding guild are intense and potentially demographically significant, and so merit further research.

## 5. Conclusions

This study demonstrates that the tephritid fly feeding guild associated with *C. funkiae* is ecologically structured and site- or habitat-dependent, even across a relatively short elevational gradient. *Chaetostomella undosa*, *Paracantha gentilis*, and *Terellia occidentalis* partition use of the same host plant across sites in ways that reflect differences in access, habitat preference, and host utilization behavior. *Chaetostomella* dominated in the talus habitat site, the typical one of *C. funkiae*, while *Paracantha* was most abundant at the forest edge site, and *Terellia* was broadly present across all three sites. Individual plant characteristics, particularly flower head diameter, emerged as an important additional predictor of fly abundance for two of the three fly species, suggesting that variation in host utilization operates at both the site and the individual plant levels. Despite near-complete seed destruction across all three sites, plants at the Forest Edge site produced significantly more viable seeds than did those in the high, open, characteristic talus habitat, a pattern associated with differences in tephritid guild composition rather than with overall fly abundance. Together, these findings indicate that tephritid herbivory on this uncommon alpine thistle is intense, microhabitat-structured, and potentially ecologically consequential. Microhabitat occupancy has meaningful implications for host plant reproductive success. Further research across multiple sites and seasons is needed to fully characterize the population-level consequences of this interaction and the mechanisms driving the observed patterns of habitat use.

## Figures and Tables

**Table 1:** Life stages of tephritid flies recorded in dissected *Cirsium funkiae* flower heads by species and site.

Species	Stage	High Site	Meadow Site	Forest Edge
*Chaetostomella undosa*	Larva	64	7	9
	Pupa	100	12	6
	Adult	0	0	2
*Paracantha gentilis*	Larva	37	133	18
	Pupa	2	1	61
	Adult	0	0	6
*Terellia occidentalis*	Larva	5	17	2
	Pupa	121	138	36
	Adult	0	6	2

Counts of all immature and adult life stages of three tephritid fly species (*Chaetostomella undosa*, *Paracantha gentilis*, and *Terellia occidentalis*) recovered from dissected *Cirsium funkiae* flower heads. Data are partitioned by developmental stage (larva, pupa, adult) across three sites: High Site, Meadow Site, and Forest Edge. Values represent individual specimens recorded during sampling, including dead or desiccated individuals. Tephritids identified by Dr. David H. Headrick of California Polytechnic State University.

## Data Availability

The original data presented in the study are openly available in FigShare at https://doi.org/10.6084/m9.figshare.33359499.

## Abbreviations

The following abbreviations are used in this manuscript:

TWI	Topographic Wetness Index
DEM	Digital Elevation Model
NLCD	National Land Cover Database
